# Risk versus Benefit of Using Hydroxychloroquine to Treat Patients with COVID-19

**DOI:** 10.1155/2021/5942366

**Published:** 2021-09-15

**Authors:** George G. Zhanel, Michael A. Zhanel, Kevin F. Boreskie, Joseph P. Lynch, James A. Karlowsky

**Affiliations:** Department of Medical Microbiology/Infectious Diseases, Max Rady College of Medicine, University of Manitoba, Winnipeg, Manitoba, Canada

## Abstract

Hydroxychloroquine (HCQ), also known by its trade name Plaquenil®, has been used for over 50 years as a treatment for malaria, systemic lupus erythematosus, and rheumatoid arthritis. As the COVID-19 pandemic emerged in the United States and globally in early 2020, HCQ began to garner attention as a potential treatment and as prophylaxis against COVID-19. Preliminary data indicated that HCQ as well as chloroquine (CQ) possessed in vitro antiviral activity against severe acute respiratory syndrome coronavirus 2 (SARS-CoV-2). Early clinical data from China and France reported that HCQ and CQ were associated with viral load reduction and clinical improvement in patients with COVID-19 compared to control groups; however, an overwhelming number of randomized controlled trials, meta-analyses, and systematic reviews have since concluded that HCQ used alone, or in combination with azithromycin (AZ), provides no mortality or time-to-recovery benefit in hospitalized patients with COVID-19. Additionally, these same trials reported adverse events including cardiac, neuropsychiatric, hematologic, and hepatobiliary manifestations in patients with COVID-19 whom had been treated with HCQ. This review article summarizes the available data pertaining to the adverse events associated with HCQ use, alone or in combination with azithromycin, in patients with COVID-19 in order to fully assess the risk versus benefit of treating COVID-19 patients with these agents. The results of this review lead us to conclude that the risks of adverse events associated with HCQ use (with or without AZ) outweigh the potential clinical benefits and thus recommend against its use in the treatment or prevention of COVID-19.

## 1. Introduction

**Figure 1:**
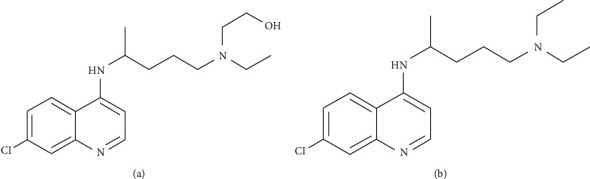
Chemical structure of (a) hydroxychloroquine (HCQ) and (b) chloroquine (CQ).

Hydroxychloroquine (HCQ), also known by its trade name Plaquenil®, received approval by the United States Food and Drug Administration (FDA) for the medical treatment of malaria in 1955 [1]. Although newer medications have largely replaced HCQ in the treatment of malaria, the drug continues to be a common pharmacological option in the treatment of chronic conditions such as systemic lupus erythematosus and rheumatoid arthritis in the United States and Canada [1]. HCQ and its sister medication, chloroquine (CQ), belong to a family of compounds known as 4-aminoquinolines based on their chemical structure [2]. The compounds are chemically identical aside from a hydroxyl group attached to one of the *N*-ethyl side chains in the HCQ molecule (Figures 1(a) and 1(b)) [2]. However, the pharmacological properties of the two drugs differ slightly. Most notably, HCQ is known to be less toxic than CQ and is associated with a reduced risk of adverse events, making it favourable in the majority of clinical settings [3]. HCQ exerts an immunomodulatory mechanism of action through a variety of mechanisms including increasing lysosomal pH, inhibiting transcription and release of cytokines, and inhibiting costimulatory molecular signaling [2]. Beyond the known antimalarial and immunomodulatory effects of HCQ, its potential to act as an anti-inflammatory and antiviral agent has recently sparked particular interest in the context of coronavirus disease (COVID-19) infections caused by the severe acute respiratory syndrome coronavirus 2 (SARS-CoV-2) [1].

The World Health Organization (WHO) declared the SARS-CoV-2 outbreak a pandemic on March 11, 2020 [4]. The vast social, economic, and public health impacts of this pandemic around the world have driven a surge in research aimed at finding effective treatments for the disease. Several agents, including HCQ, garnered global interest as potential treatments for COVID-19. On March 28, 2020, the FDA approved emergency use authorization of HCQ and CQ for the treatment of COVID-19 [5]. On June 15, 2020, the FDA then revoked this emergency use authorization for both HCQ and CQ as emerging literature, including the influential RECOVERY trial, indicated no clinical benefit attributed to HCQ use in the treatment of COVID-19 [6]. To date, numerous randomized controlled trials, meta-analyses, systematic reviews, and opinions have been published evaluating the efficacy and safety of HCQ used alone, or in combination with azithromycin (AZ), in the treatment of COVID-19 or as a prophylactic agent against the virus [6–40]. The overwhelming conclusion from all these data is that HCQ with or without AZ does not provide a mortality or time-to-recovery benefit in hospitalized patients with COVID-19 [6, 7]. The question that remains is what data exist regarding the adverse events of HCQ in patents treated for COVID-19.

HCQ is administered differently when treating chronic conditions as compared to COVID-19. For chronic conditions, such as rheumatoid arthritis, HCQ is commonly prescribed at a daily dose of 200–400 mg after obtaining a good response from a loading dose of 400–600 mg daily [8]. Treatment duration can last months to years in patients with autoimmune diseases. Doses as high as 800 mg per day have been reported in the treatment of malaria, although treatment duration is short (i.e., days) in these cases [8]. For treating patients with COVID-19, a wide range of dosing regimens has been used including daily doses ranging from 200 to 1000 mg daily with loading doses as high as 1200 mg in some cases; albeit treatment courses typically last for days in these patients as opposed to months or years [9].

HCQ which is primarily excreted in the urine (as unchanged drug or as metabolites) is reasonably well tolerated in most patients at standard doses [10–12] with gastrointestinal side effects including nausea, vomiting, diarrhea, anorexia, and abdominal pain being most common (Table 1) [10–12]. However, serious cardiac, neurologic, psychiatric, hepatobiliary, and hematologic adverse events have been associated with HCQ and CQ in patients with COVID-19 as well as in other patient populations (Table 1) [9–13]. Certain ocular manifestations, dermatologic reactions, and other adverse events have additionally been documented in association with these agents in non-COVID-19 patients [10–12].

This review article focuses on the risk of using HCQ alone, or in combination with AZ, in patients with COVID-19. Where possible, we have specified the severity of disease in the study population. In areas where limited data are available in COVID-19 patients, we have commented on the toxicity of HCQ in other patient populations. This review which focused on articles published in 2020 and 2021 (on PubMed) serves to answer whether the risk of using HCQ, with or without AZ, in treating patients with COVID-19 outweighs the potential benefit.

## 2. Cardiac Toxicity

Adverse cardiac events, including conduction abnormalities, cardiomyopathy, and sudden cardiac death, are rare, yet known and labeled potential adverse events of HCQ and CQ use (Table 1) [8]. Given the potential for COVID-19 to be associated with cardiac complications independent of pharmacological intervention, it is incumbent to explore the risk of HCQ treatment, especially in critically ill patients with COVID-19 [14, 15]. The available literature citing cardiac adverse events associated with HCQ or CQ monotherapy, or in combination with AZ, in the treatment of COVID-19 has been summarized below.

On May 19, 2020, the FDA published a pharmacovigilance memorandum providing a safety overview of the use of HCQ and CQ in patients with COVID-19 [9]. Data were compiled from several databases including the FDA Adverse Event Reporting System (FAERS) database, the American Association of Poison Control Centers National Poison Data System (AAPPC-NPDS), published medical literature, and other safety reports forwarded from the Division of Antiviral (DAV) drug products. Between December 1, 2019, and May 6, 2020, a total of 347 cases of adverse events related to HCQ and 38 adverse events related to CQ were reported in patients with COVID-19. Serious cardiac adverse events associated with HCQ were documented in 90 of these cases. Of these, 62 cases were reports of QT prolongation. Eleven patients experienced ventricular arrhythmia (VA), ventricular fibrillation (VF), or ventricular tachycardia (VT). Four cases of Torsades de Pointes (TdP) were reported. Other documented cardiac adverse events included bradycardia, tachycardia, arrhythmias (excluding VA, VF, and VT), AV block, and QRS prolongation. Atrial fibrillation/atrial flutter and myocardial infarction were reported as unlabeled cardiac adverse events. The most common concomitant treatment agent was AZ, which was documented in 55 cases, highlighting the potential importance of AZ in contributing to toxicity.

Cardiac toxicity has been documented in several systemic reviews and meta-analyses, which have assessed the safety and efficacy of HCQ and CQ in the treatment of patients with COVID-19. On February 5, 2021, the Mayo Clinic published a systematic review and metaregression analysis of cardiac toxicity associated with HCQ or CQ, alone or in combination with AZ, in patients with COVID-19 [15]. This review incorporated 5652 participants across 19 studies published between November 2019 and May 27, 2020. The pooled incidence of TdP, ventricular tachycardia, and cardiac arrest was 3 per 1000 (95% CI, 0–21; *I*^2^ = 96%) in 18 studies with 3725 participants. Among 13 studies of 4334 patients, the pooled incidence of discontinuation of CQ or HCQ due to prolonged QTc or arrhythmias was 5 per 100 (95% CI, 1–11; *I*^2^ = 98%). The pooled incidence of change in QTc from baseline of 60 milliseconds or more, or QTc of 500 milliseconds or more was 9 per 100 (95% CI, 3–17; *I*^2^ = 97%).

Based on the available data, HCQ used alone or in combination with AZ in the treatment of patients with COVID-19 has been associated with statistically significant prolongation in QTc intervals [9, 15]. HCQ in combination with AZ may result in greater QTc prolongation when compared to HCQ monotherapy [9, 15]. QTc prolongation has been observed at a variety of HCQ doses in available studies, generally ranging from daily doses of 200 mg to 800 mg with treatment durations commonly lasting 5–10 days [9, 15]. Although it seems that conduction abnormalities including TdP in these patients are rare, more data are needed to quantitate this risk. A case report of a patient suffering from ventricular fibrillation and cardiac arrest due to QTc prolongation from a combination of HCQ/AZ has been documented (Table 1) [12]. As growing evidence suggests that COVID-19 may independently be associated with cardiotoxicity, especially in older patients and in patients with preexisting comorbidities, cardiac monitoring is advised if HCQ is being used alone and especially if used in combination with AZ in patients with COVID-19 [12, 13].

## 3. Neuropsychiatric Adverse Events

Neuropsychiatric adverse events are known and labeled adverse events associated with HCQ and CQ use [8]. Numerous case reports have identified a wide range of psychiatric manifestations associated with acute and chronic use of these agents [17–22]. Exacerbations of preexisting mental health diagnoses including psychosis, depression, and bipolar disorder have been documented [17–22]. New-onset psychiatric events have also been reported in all age groups and with treatment durations as short as 24 hours [23]. A recent retrospective study published by Sato et al. identified 520 neuropsychiatric adverse events associated with CQ [22]. These researchers reported increased prevalence of amnesia, delirium, hallucinations, depression, and loss of consciousness associated with CQ use [22]. Additionally, several case reports have implicated HCQ in triggering generalized tonic-clonic seizures in nonepileptic patients [24, 25]. In these cases, treatment durations ranging from 2 to 6 weeks at standard doses were sufficient to trigger seizure activity. The FDA has reported five cases of hallucinations or psychosis attributed to HCQ or CQ in patients with COVID-19 [9]. Two reports of seizures attributed to CQ in patients with COVID-19 have also been identified by the FDA [9]. Recent literature has suggested that encephalitis is possible sequelae of COVID-19 [26, 27]. Given the risk for HCQ and CQ to result in neuropsychiatric adverse events, caution should be exercised when using these agents in patients with COVID-19 until further data are available to quantitate this risk.

## 4. Hematologic Adverse Events

A variety of hematological consequences may occur as a result of COVID-19 including lymphopenia, which may even be a cardinal prognostic indicator in critically ill patients [28]. Prolongation of coagulation markers including prothrombin time (pT) and partial thromboplastic time (aPPT) has been documented in patients with COVID-19 [28]. Severe thrombocytopenia, and in some cases disseminated intravascular coagulation (DIC), as sequelae of COVID-19, has been proven deadly [28]. Generally, HCQ has been known to have little influence on hematologic parameters, although case reports citing anemia, agranulocytosis, leukopenia, and thrombocytopenia have been published (Table 1). The drug insert for Plaquenil® cautions against its use in patients with G6PD deficiency [8]. To date, the FDA has reported 17 cases of hematologic adverse events associated with HCQ in patients with COVID-19 [9]. Of these, five cases of hemolytic anemia related to G6PD deficiency have been identified [9]. Thrombocytopenia, anemia, leukopenia, or pancytopenia was reported in 12 cases [9]. One case of agranulocytosis has been documented [9].

## 5. Hepatobiliary Manifestations

The drug insert for Plaquenil® suggests exercising caution in using this agent in patients with hepatic disease, alcoholism, or concomitant use of known hepatotoxic medications [8]. Cavalcanti et al. conducted a multicenter, randomized, open-label, three-group, controlled trial assessing the safety and efficacy of HCQ with or without azithromycin in patients with mild or moderate cases of COVID-19 [29]. They reported increased liver enzyme concentration in patients treated with HCQ alone, or in combination with azithromycin, compared to subjects not receiving these agents [29]. The FDA has reported a total of 67 cases of hepatitis, transaminitis, hyperbilirubinemia, or liver failure related to HCQ or CQ use in patients with COVID-19 [9]. As new literature emerges identifying hepatobiliary toxicity attributed to HCQ or CQ, with or without AZ, in patients with COVID-19, it is important to recognize the risk of these agents in this patient population.

## 6. Ocular Toxicity

HCQ-induced retinopathy is a well-recognized phenomenon, and the risk of retinal toxicity increases with increased duration and dosage of HCQ treatment [30]. Petri et al. conducted a large cohort study including 537 patients with systemic lupus erythematosus treated with HCQ [30]. They reported the risk of retinopathy after five years of treatment to be 1%, 1.8% up to 10 years of treatment, 3.3% up to 15 years, and 11.5% up to 20 years of treatment [30]. These authors reported the overall frequency of retinopathy to be 4.3% in this patient cohort [30]. Patients with older age, higher body mass index, longer duration of treatment, and higher serum concentrations of HCQ had increased risk of developing retinopathy [30]. The American Academy of Ophthalmology (AAO) suggests not exceeding doses of 5 mg/kg/day in order to limit the risk of retinopathy [30]. At this time, to the best of our knowledge, data have not been published assessing the incidence of retinopathy in patients with COVID-19 treated with HCQ. Considering that treatment durations used in patients with COVID-19 are generally much shorter than those used in patients with autoimmune conditions such as systemic lupus erythematosus, the risk of retinopathy is likely significantly lower in this population. Nevertheless, ophthalmological screening for retinopathy should be a consideration, especially in patients taking HCQ for an extended period of time to treat COVID-19.

## 7. Dermatologic Manifestations

Dermatologic reactions associated with HCQ have been recognized for decades with its use as an antimalarial and immunologic agent, but very limited data exist in patients treated with HCQ for COVID-19. A recent systematic review published by Sharma et al. outlined the most common dermatologic reactions associated with HCQ [31]. This systematic review summarized data from 94 articles, citing a total of 689 adverse dermatologic reactions associated with HCQ [31]. The authors noted that, in many cases, the reactions were poorly characterized, with unknown frequencies and risk factors [31]. Over 20 unique dermatologic reactions were recorded, most commonly drug eruption or rash (358 cases), cutaneous hyperpigmentation (116 cases), pruritis (62 cases), acute generalized exanthematous pustulosis (27 cases), Stevens–Johnson syndrome or toxic epidermal necrolysis (26 cases), hair loss (12 cases), and stomatitis (11 cases) [31]. The most common indications for use of HCQ in these studies were for patients with systemic lupus erythematosus (74% of patients) and rheumatoid arthritis (14% of patients) [31]. Dermatologic manifestations were observed at a wide range of mean cumulative doses with evidence suggesting they could occur at a cumulative dose as low as 3 grams [31]. Based on common dosing regimens used in COVID-19 patients, it is reasonable to expect that dermatologic reactions may arise in patients undergoing treatment with HCQ for COVID-19. To date, the FDA has documented one case of an exacerbation of psoriasis attributed to CQ use in a patient with COVID-19 [9]. Careful monitoring in these patients is advised until further data is available in this patient population.

## 8. Glucose Metabolism and Other Adverse Events

The FDA has reported a variety of adverse events attributed to HCQ or CQ in patients with COVID-19 that are not identified on the label of these agents [9]. Among these, adverse events include hyperglycemia as well as acute kidney injury or renal failure, rhabdomyolysis, methemoglobinemia, hypokalemia, hyponatremia, oropharyngeal edema, and anuria [9]. COVID-19 patients with chronic medical comorbidities including diabetes, cardiovascular disease, chronic lung disease, chronic kidney disease, and cancer are at an increased risk of morbidity and mortality [32]. The effects of COVID-19 on glucose metabolism specifically are controversial [32]. Some reports have suggested that patients with COVID-19 are likely to experience hyperglycemia due to catecholamine and glucocorticoid release during acute illness [32]. Conversely, a study from Wuhan, China reported that at least 10% of COVID-19 patients with type-2 diabetes mellitus experienced at least one episode of hypoglycemia [32]. The use of concurrent pharmacologic agents in managing both acute illness and chronic diseases in the context of COVID-19 further complicates this issue. At this time, no studies have reviewed the influence of HCQ on glucose plasma concentrations in patients with COVID-19. HCQ has long been known to possess hypoglycemic properties [33]. In a systematic review summarizing data from 55,776 study participants, Wondafresh et al. presented data showing the ability of HCQ to increase insulin sensitivity, decrease HbA1c levels, decrease fasting plasma glucose levels, and decrease postprandial glucose levels [34]. Case reports have documented life-threatening hypoglycemic events associated with HCQ (Table 1) [35, 36]. Given the potential for HCQ use to be associated with hypoglycemia, patients treated with this agent in the context of COVID-19 should have plasma glucose concentrations monitored routinely especially if they have diabetes. In addition, medication review and consolidation are recommended in diabetic patients with COVID-19 in order to avoid episodes of hypoglycemia.

## 9. Summary of HCQ Adverse Events in Patients with COVID-19

Based on available literature, HCQ-associated QTc prolongation is a common adverse event in patients with COVID-19 treated with this agent. When used in combination with AZ, this risk appears to be increased. Severe QTc prolongation (i.e., >500 ms or QTc prolongation requiring cessation of treatment) is also a common occurrence. The risk of arrhythmias, including TdP and ventricular fibrillation, is poorly quantitated at this time due to the scarcity of reports, although the risk appears to be rare based on available data. The risk of cardiomyopathy in association with HCQ remains poorly understood and has yet to be documented in patients with COVID-19 treated with this agent. Various neuropsychiatric manifestations associated with HCQ have been documented in patients treated for indications including malaria, rheumatoid arthritis, and systemic lupus erythematosus. Psychosis, hallucinations, and seizures have been reported in patients with COVID-19 treated with these agents. Hematologic manifestations including hemolytic anemia in patients with G6PD deficiency, thrombocytopenia, lymphopenia, and agranulocytosis have been documented in patients treated with HCQ for COVID-19. Likewise, hepatobiliary manifestations have been observed in COVID-19 and non-COVID-19 patients treated with these agents. Ocular and dermatologic manifestations due to HCQ have so far been poorly documented in patients treated for COVID-19.

## 10. Conclusion

In this review article, we have summarized the available data pertaining to the adverse events associated with HCQ use, alone or in combination with azithromycin, in patients with COVID-19 in order to fully assess the risk versus benefit of treating COVID-19 patients with these agents. The results of this review lead us to conclude that the risk of adverse events associated with using hydroxychloroquine (with or without azithromycin) outweigh the potential clinical benefits and thus recommend against its use in the treatment of COVID-19.

## Figures and Tables

**Table 1:** Frequency of adverse drug events associated with hydroxychloroquine in COVID-19 and non-COVID-19 patients.

Adverse event type	Described events	Frequency in COVID-19 patients^a,b^	Frequency in non-COVID-19 patients^a^	References
Cardiac	QTc prolongation^c^	Common	Well documented	[8–15, 40]
	Severe QTc prolongation^d^	Common	Well documented	
	Arrhythmias (TdP)	Rare	Case reports	
	Ventricular tachycardia	Rare	Case reports	
	Cardiomyopathy	—	Case reports	
	Cardiac death	Case report	Case report	
	Sinus arrest	Rare	Case report	

Neuropsychiatric	Headache	—	Common	[8, 9, 17–27]
	Mood changes	—	Common	
	Psychosis	Case reports	Case reports	
	Depression	—	Case reports	
	Bipolar disorder	—	Case reports	
	Amnesia	—	Case reports	
	Delirium	—	Case reports	
	Hallucinations	Case reports	Case reports	
	Depression	—	Case reports	
	Loss of consciousness	—	Case reports	
	Seizures	Case reports	Case reports	

Hematologic	Hemolytic anemia in G6PD	Case reports	Drug warning	[8, 9, 28]
	Agranulocytosis	Case reports	Case reports	
	Thrombocytopenia	Case reports	Case reports	
	Leukopenia	Case reports	Case reports	

Hepatobiliary	Hepatitis/↑LEs^e^/↑bilirubin	Case reports	Drug warning	[8, 9, 29]
	Hepatic failure	Case reports	—	

Ocular	Retinopathy	—	Common	[8, 9, 30]

Dermatological	Exacerbation of psoriasis	Case report	Case reports	[8, 9, 31]
	Drug eruption/rash	—	Common	
	Pruritus	—	Common	
	Hyperpigmentation	—	Case reports	
	AGEP^f^	—	Case reports	
	TEN^g^	—	Case reports	
	SJS^h^	—	Case reports	
	Hair loss	—	Case reports	
	Stomatitis	—	Case reports	

^a^Frequency of adverse drug reactions reported using the Council of International Organization Medical Sciences (CIOMS) scale: very common, ≥1/10; common, ≥1/100 and <1/10; uncommon, ≥1/1,000 and <1/100; rare, ≥1/10,000 and <1/1,000; and very rare, <1/10,000. ^b^Based on available literature that may be inconclusive/incomplete. ^c^QTc prolongation defined as significant increase from baseline QTc interval. ^d^Severe QTc prolongation defined as QTc interval >500 ms or QTc prolongation requiring discontinuation of treatment. ^e^LE, liver enzymes. ^f^AGEP, acute generalized exanthematous pustulosis. ^g^TEN, toxic epidermal necrolysis. ^h^SJS, Stevens–Johnson syndrome.

## Data Availability

Data are available from PubMed search.

## Additional Points

*Highlights.* (1) Hydroxychloroquine-associated QTc prolongation is a common adverse event in patients with COVID-19 treated with this agent and when used in combination with AZ, this risk appears to be increased. (2) Various neuropsychiatric, hematologic, hepatobiliary, ocular, and dermatologic manifestations as well as glucose metabolism alterations due to hydroxychloroquine have been poorly documented in patients treated for COVID-19. (3) The risks of adverse events associated with hydroxychloroquine use (with or without azithromycin) outweigh the potential clinical benefits, and thus, we recommend against its use in the treatment or prevention of COVID-19.
